# Management of Giant Recurrent Retrosternal Thyroid Goiter in a Low-Volume Surgical Center: A Case Report

**DOI:** 10.7759/cureus.100194

**Published:** 2025-12-27

**Authors:** Qahtan A Al Dulaimi, Mais O Abu-Sa'da, Rand N Fatayerji, Ahmad Alaboud, Fadi Al Masalmeh

**Affiliations:** 1 General Surgery, Saqr Hospital, Emirates Health Services, Ras Al-Khaimah, ARE; 2 General Surgery, RAK Medical and Health Sciences University, Ras Al-Khaimah, ARE; 3 Medicine, Ibrahim Bin Hamad Obaidallah Hospital, Emirates Health Services, Ras Al-Khaimah, ARE

**Keywords:** enlarged thyroid, euthyroid goiter, multinodular goiter, neck swelling, retrosternal goiter, thyroid goiter, thyroid surgery, tracheal deviation

## Abstract

Retrosternal goiters may extend beneath the sternum and exert pressure on surrounding structures as they enlarge. Recurrent retrosternal goiters present additional surgical challenges because previous operations can alter normal anatomic planes. We report the case of a 53-year-old woman who presented to our hospital with a gradually enlarging anterior neck mass approximately 16 years after prior cervical surgery. Operative records from the earlier procedure were unavailable, and the nature of the prior surgery could not be determined. She denied stridor, dyspnea, orthopnea, or voice changes. Imaging demonstrated a large multinodular goiter with retrosternal extension to the level of the thoracic inlet, causing tracheal displacement without significant luminal narrowing. Thyroid function was normal, and fine needle aspiration showed benign cytology. The patient underwent total thyroidectomy through a standard cervical collar incision, without the need for sternotomy. Intraoperatively, overlying soft tissue adhesions and scarring were noted. The retrosternal component was removed safely through the cervical approach. The excised specimen weighed approximately 410 g. The right lobe measured 14 × 8.5 cm, the left lobe 8.5 × 3.5 × 4 cm, and the isthmus 2 × 2 cm. Both recurrent laryngeal nerves were preserved, parathyroid function remained intact, and the postoperative course was uncomplicated with complete symptom resolution. Histopathologic examination confirmed benign multinodular hyperplasia with degenerative changes, without evidence of malignancy. This case highlights that even large recurrent retrosternal goiters with limited intrathoracic extension can often be safely managed via a cervical approach, emphasizing the value of careful preoperative assessment and surgical planning.

## Introduction

Retrosternal goiters are enlargements of the thyroid gland that extend into the mediastinum and represent a recognized subset of thyroid disease. Their descent into the thoracic cavity is clinically significant because it may result in tracheal deviation, dysphagia, or vascular compression. Most retrosternal goiters are benign multinodular enlargements, but deep extension can create diagnostic and operative challenges, particularly when the goiter becomes large [1].

Patients may present with compressive symptoms such as dyspnea, orthopnea, or difficulty swallowing, although some remain asymptomatic until the mass reaches a considerable size [1]. Surgical management can be demanding due to limited operative space, distorted anatomy, adhesions and scarring, and proximity to vital mediastinal structures, which increases the technical complexity of resection [2].

Recurrent retrosternal goiters are uncommon, and prior thyroid surgery further complicates operative planning because scarring and altered anatomy heighten the risk of injury to the recurrent laryngeal nerves and parathyroid glands [2]. Giant recurrent retrosternal goiters without airway compromise are particularly rare. We report such a case successfully managed through a cervical approach, highlighting key operative considerations and outcomes while contributing to the surgical literature.

## Case presentation

History and physical examination

A 53-year-old woman presented to our hospital with a progressively enlarging anterior neck swelling over a two-year period. The enlargement was more pronounced on the right side and developed gradually without symptoms of hyperthyroidism or hypothyroidism. She denied dysphagia, shortness of breath, orthopnea, voice changes, or any sensation of airway pressure. Her review of systems was otherwise unremarkable. Past medical history was non-significant, and her only prior surgery was a thyroid procedure in 2008. She reported a family history of thyroid disease in second-degree relatives.

**Figure 1:**
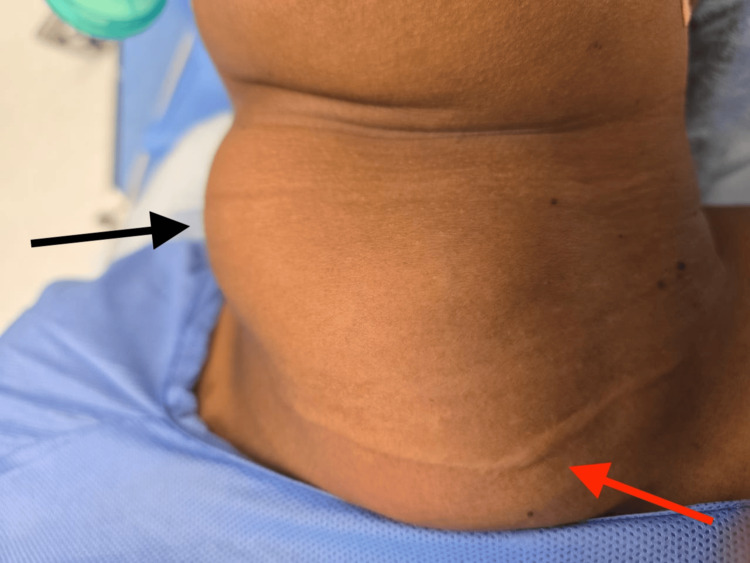
Visibly enlarged thyroid on physical examination represented by black arrow. Well-healed transverse surgical scar at lower neck from previous thyroid surgery represented by red arrow.

On physical examination, the patient was breathing comfortably with no stridor. A large multinodular thyroid enlargement was observed, predominantly involving the right lobe (Figure 1). The mass moved with swallowing and was not adherent to the overlying skin or underlying structures. The right lobe extended retrosternally. The nodules were firm and non-tender, and no cervical lymphadenopathy was detected. Pemberton's sign was negative. A well-healed transverse surgical scar was noted in the lower neck (Figure 1).

Airway assessment was unremarkable. Flexible laryngoscopy confirmed normal vocal cord structure and motion. Thyroid function tests were within normal limits (Table 1).

**Table 1:** Laboratory results for the patient on the day of surgery BUN: blood urea nitrogen; INR: international normalised ratio; PTT: partial thromboplastin time; eGFR: estimated glomerular filtration rate; ALT: alanine transaminase; AST: aspartate aminotransferase; ALP: alkaline phosphatase; MCV: mean corpuscular volume; T4 free: free thyroxine; TSH: thyroid stimulating hormone

Tests	Patient Value	Reference Range
BUN	2.5 mmol/L	2.1 - 8.5 mmol/L
Calcium Lvl	2.4 mmol/L	2.20-2.60 mmol/L
Chloride lVl	105 mmol/L	98-107 mmol/L
CO2	29.0 mmol/L	22-29 mmol/L
Creatinine	62.0 umol/L	44-80 umol/L
Hematocrit	42.6 %	36-46 %
Hemoglobin	13.8 g/dL	12-15.5 g/dL
INR	0.93 ratio	0.8-1.2 ratio
Platelet	287.00 ×10³/µL	150 – 400 ×10³/µL
Potassium level	4.6 mmol/L	3.5-5.1 mmol/L
PTT	38.30 Second(s)	25-35 Second (s)
Sodium level	144 mmol/L	135-145 mmol/L
WBC	4.62 ×10³/µL	4.0-11.0 ×10³/µL
eGFR	104 mL/min/1.73 m²	More than 90 Normal
Total Protein	76 gm/L	60-80 g/L
Albumin Lvl	39 gm/L	35-50 g/L
Bilirubin Total	5 umol/L	< 21 umol/L
ALT	18 U/L	< 40 U/L
AST	13 U/L	< 40 U/L
ALP	64.0 U/L	40-120 U/L
RBC	5.2 ×10⁶/µL	4.2-5.4 ×10⁶/µL
MCV	81.70 fL	80-100 fL
T4 Free	11.78 pmol/L	10-22 pmol/L
TSH	0.62 mIU/L	0.4-4.0 mIU/L

Imaging

**Figure 2:**
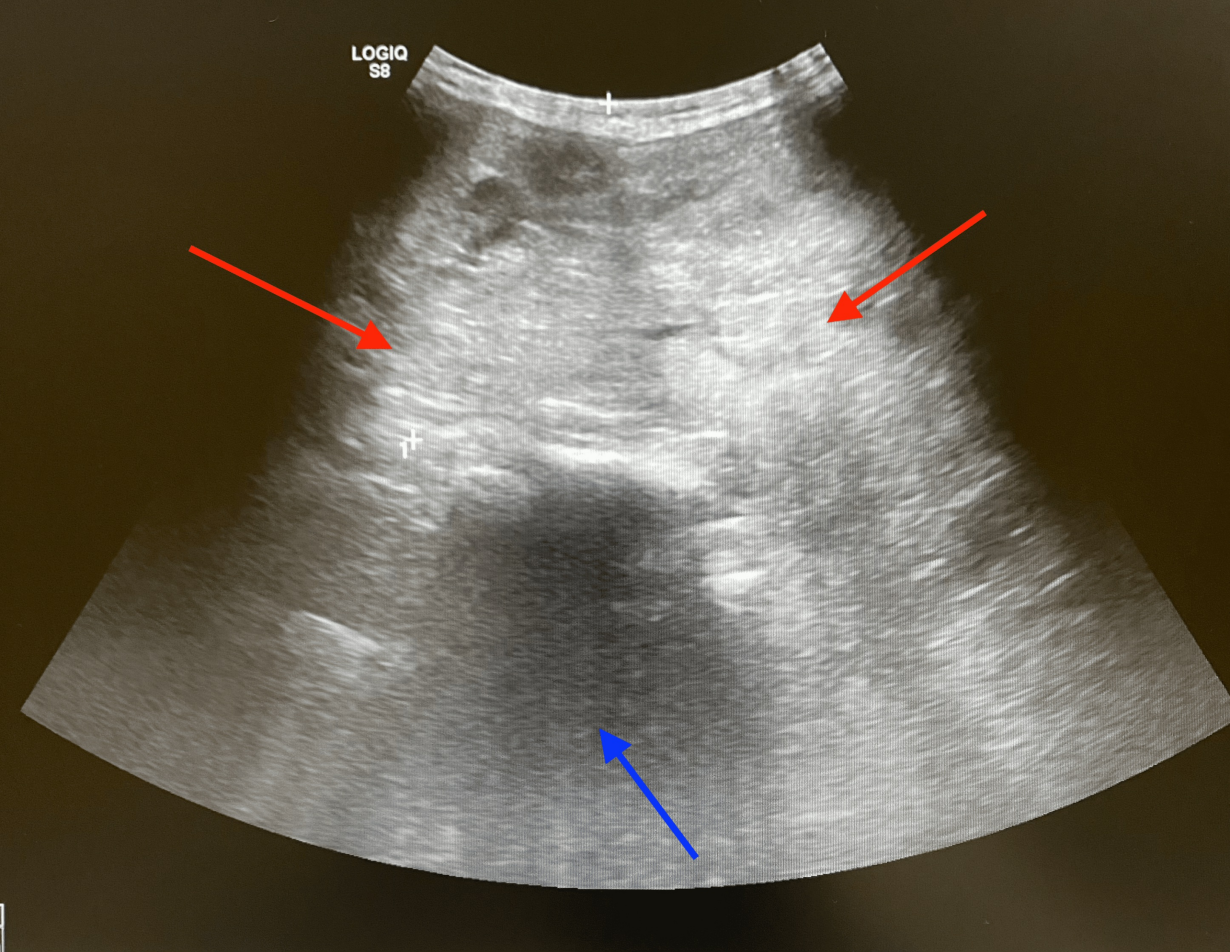
Ultrasound of the neck in the transverse plane showing markedly enlarged and heterogeneous thyroid tissue consistent with goiter (red arrows). The hypo-echoic trachea is displaced leftward by the enlarged right thyroid lobe (blue arrow).

Ultrasound demonstrated enlargement of both thyroid lobes, with a markedly enlarged right lobe containing multiple nodules of varying size and showing retrosternal extension (Figure 2). Fine needle aspiration from both lobes yielded benign cytology consistent with Bethesda category two.

**Figure 3:**
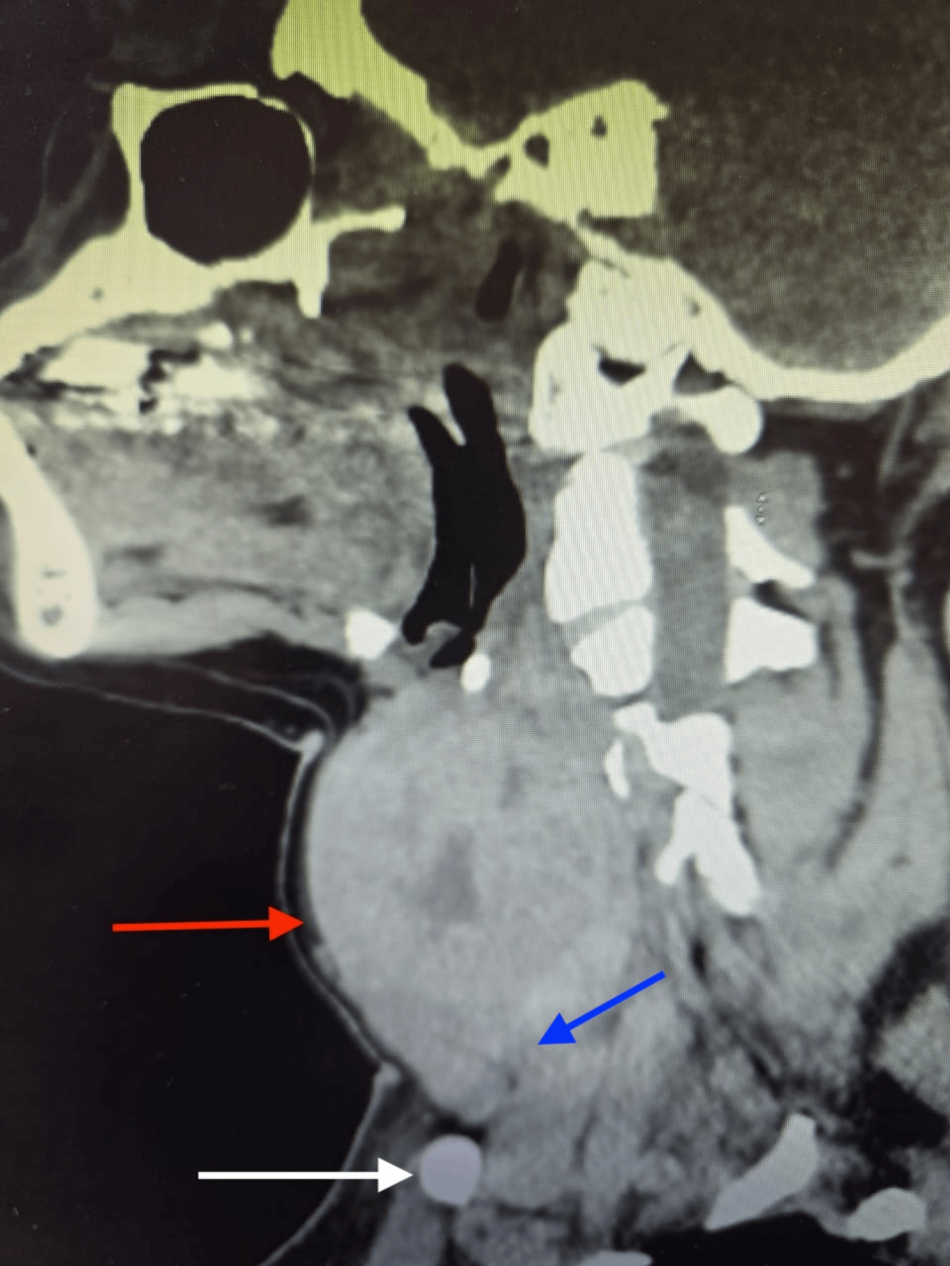
Sagittal non-contrast CT image of the neck demonstrating a markedly enlarged thyroid gland. The giant thyroid enlargement is identified occupying the lower cervical region (red arrow). Inferior extension of the thyroid gland into the retrosternal space is demonstrated, consistent with retrosternal extension (blue arrow). The clavicle is visualized as a bony landmark anteriorly (white arrow).

**Figure 4:**
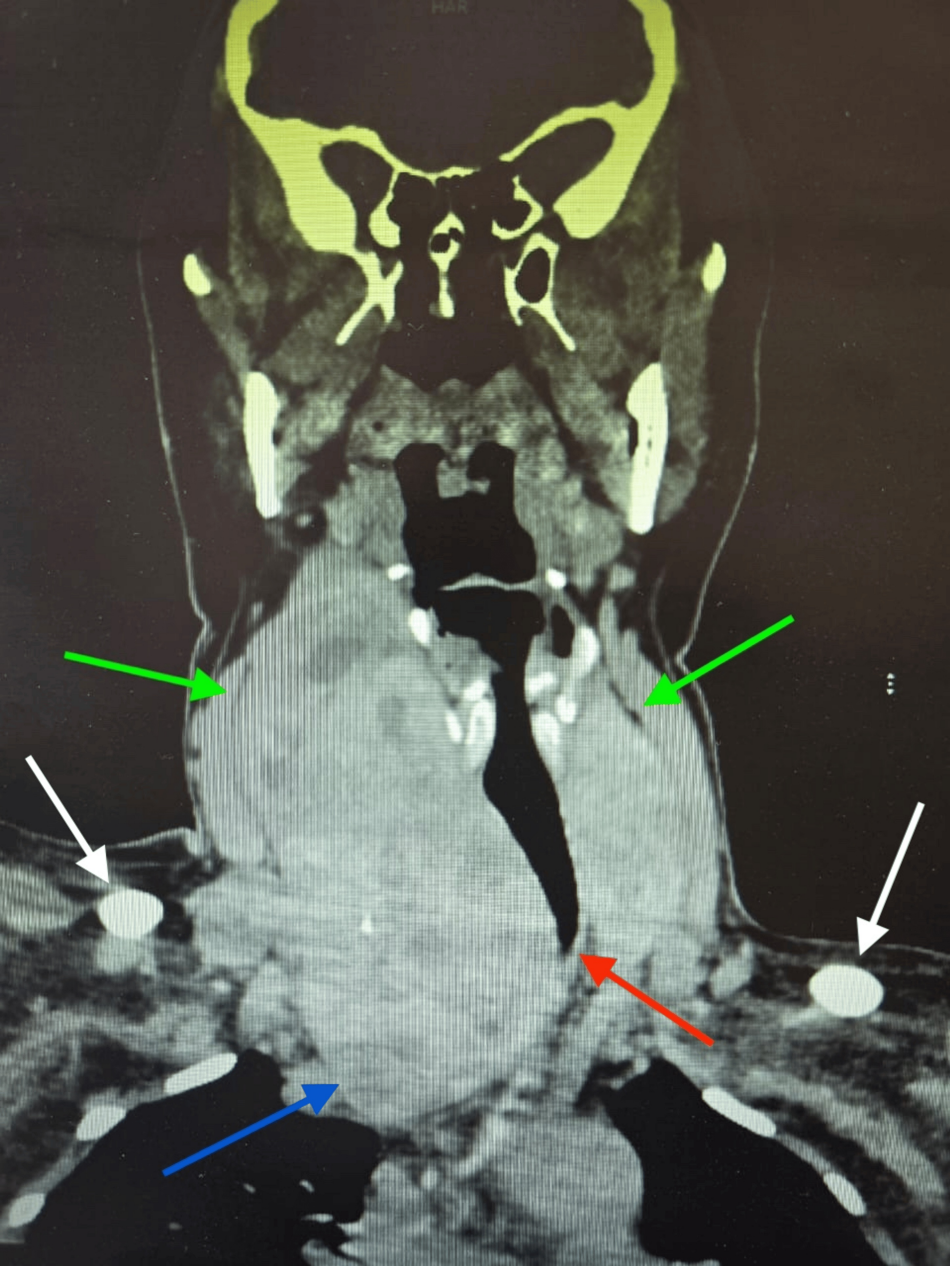
Coronal CT image of the neck without contrast demonstrating a markedly enlarged thyroid gland with inferior retrosternal extension reaching just above the confluence of the brachiocephalic veins, without significant compression of these vessels (blue arrow). The common carotid arteries are indicated (green arrows). Leftward tracheal deviation caused by the massively enlarged right thyroid lobe is noted (red arrow). The clavicles are identified (white arrows). Despite tracheal deviation, no significant airway compromise is evident on imaging.

Computed tomography (CT) of the neck showed diffuse thyroid enlargement with heterogeneous parenchyma (Figure 3). The right lobe extended inferiorly to the thoracic inlet, reaching the level just above the confluence of the brachiocephalic veins, without significant compression of these vessels. There was leftward tracheal deviation with mild narrowing, although the airway remained patent (Figure 4). The exact percentage of reduction in the diameter was not quantified. Several small bilateral cervical lymph nodes were present and appeared reactive.

Diagnosis

Based on the combined clinical and radiological findings, a diagnosis of a giant recurrent retrosternal multinodular goiter was established, and the patient was scheduled for total thyroidectomy.

Surgical management

**Figure 5:**
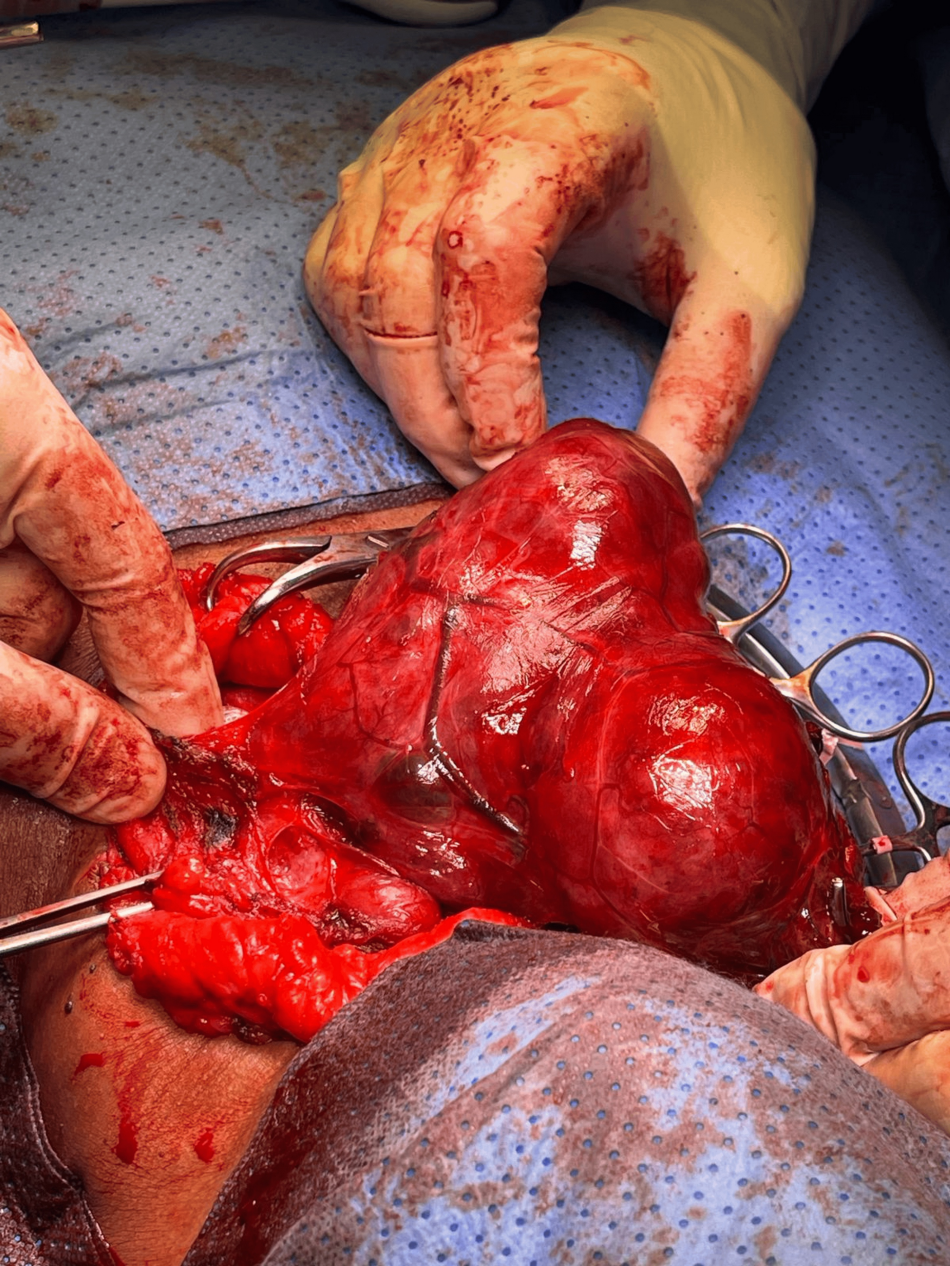
Intraoperative view of the cervical thyroidectomy. The thyroid gland is exposed through a standard cervical incision, with overlying skin and subcutaneous tissues retracted.

**Figure 6:**
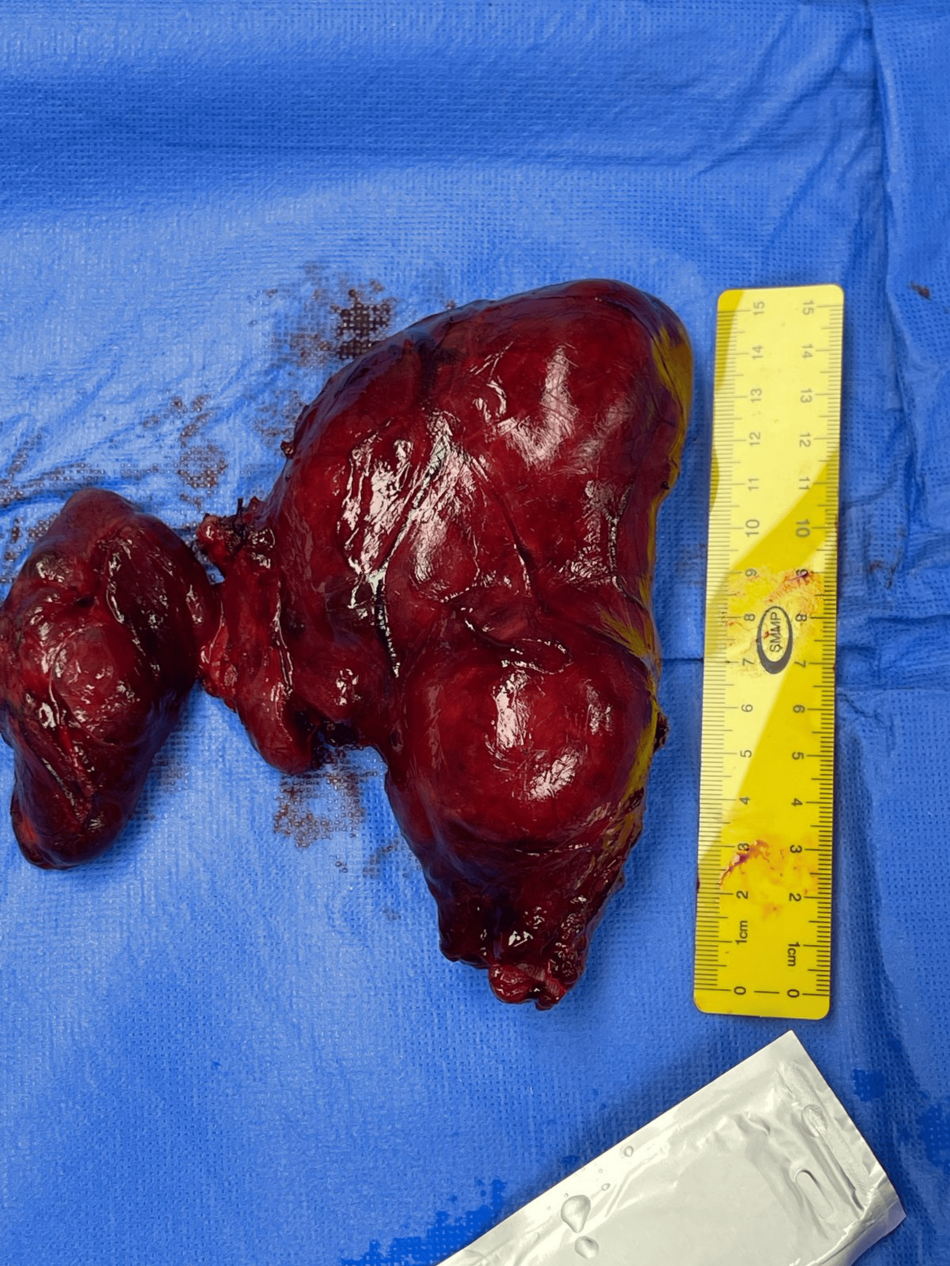
Resected enlarged thyroid gland. The specimen weighed approximately 410 g. The right lobe measured 14 × 8.5 cm, and the left lobe measured 8.5 × 3.5 × 4 cm. The isthmus measured 2 × 2 cm.

The patient underwent a total thyroidectomy through a cervical approach (Figure 5). Intraoperatively, overlying scarring and adhesions were noted, consistent with a history of previous thyroid surgery, confirming this as a case of recurrent goiter. Both thyroid lobes and the isthmus were removed (Figure 6). The thyroid gland was markedly enlarged, with retrosternal extension predominantly on the right side, but no intraoperative features suggestive of malignancy were observed.

The recurrent laryngeal nerves were identified in their normal anatomical positions and preserved bilaterally. The parathyroid glands were carefully protected to maintain normal calcium balance, and the paramedian lobe was excised. Hemostasis was secured with an estimated blood loss of approximately 50mm, and a 16 French vacuum-assisted closure (VAC) drain was placed to reduce the risk of postoperative fluid collection.

The retrosternal component was managed entirely via the cervical approach. Control of the inferior thyroid vessels was achieved from above. After complete cervical mobilization of the thyroid, digital blunt dissection (“Blount’s finger delivery”) was performed by inserting a finger behind the sternum to gently free the inferior portion of the goiter, which was then delivered upward into the neck without the need for a sternotomy.

The patient was transferred from the recovery unit in stable condition. Her postoperative course was uncomplicated, with adequate pain control and no anesthesia-related concerns. She exhibited no airway obstruction, bleeding, or changes in voice. Calcium levels remained within the normal range, confirming preserved parathyroid function. Final pathology revealed benign multinodular thyroid tissue with no evidence of malignancy.

A cervical approach was sufficient for complete removal of the retrosternal goiter while preserving the recurrent laryngeal nerves and parathyroid glands. This case highlights the surgical strategies required for recurrent goiter, including careful identification of scarred planes, safe mobilization of retrosternal tissue, and preservation of vital structures.

Pathology

**Figure 7:**
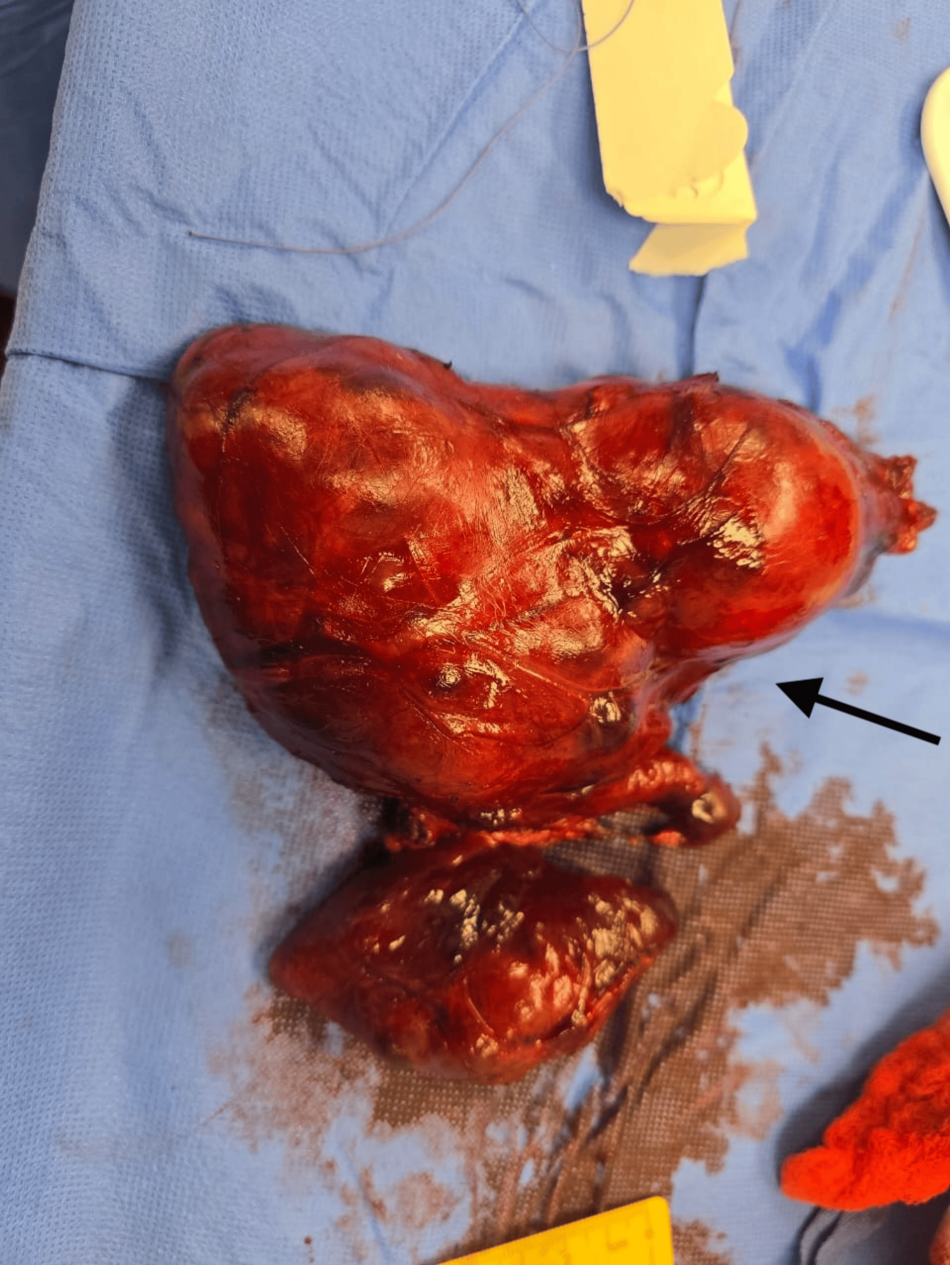
Total thyroid gland with pyramidal lobe, gross specimen. Specimen was sent for pathology, which confirmed benign multi-nodular goiter.

The total thyroidectomy specimen weighed approximately 410 g. The right lobe measured 14 × 8.5 cm, and the left lobe measured 8.5 × 3.5 × 4 cm. The isthmus measured 2 × 2 cm. The external surface appeared enlarged and nodular with areas of cystic change and old hemorrhage. The cut surface showed multiple nodules of variable size filled with brown gelatinous colloid material, along with focal calcifications. The left lobe contained a large colloid-filled cyst measuring 6 × 3 × 2.5 cm. These features were consistent with a longstanding multinodular goiter demonstrating cystic degeneration, hemorrhage, and fibrosis. The entire specimen was submitted for microscopic evaluation in 51 tissue blocks (Figure 7).

Histologic sections showed a diffusely enlarged gland with multiple nodules composed of variable-sized follicles. Some follicles contained colloid with flattened epithelium, while others showed epithelial hyperplasia and microfollicular architecture. Secondary changes were prominent and included areas of recent and old hemorrhage, hemosiderin-laden macrophages, calcification, edema, necrosis, granulomatous reaction, and stromal fibrosis. There was no capsular invasion, vascular invasion, atypical mitosis, or nuclear features suggestive of papillary carcinoma. A single parathyroid tissue fragment and one reactive lymph node were present within the surrounding adipose tissue.

The findings supported the diagnosis of benign multinodular hyperplasia of the thyroid gland with degenerative changes and no evidence of malignancy.

Follow-up

At postoperative follow-up visits, the patient demonstrated a smooth recovery with no complications. She reported normal breathing, swallowing, and voice quality. Clinical assessment confirmed there were no signs of recurrent laryngeal nerve injury, and she had no hoarseness or vocal changes. Serum calcium levels remained stable, indicating preserved parathyroid function. The surgical incision healed well with an acceptable cosmetic result, and there was no evidence of hematoma, seroma, or wound infection. She remained asymptomatic, with no recurrent neck swelling or compressive symptoms, and overall recovery was consistent with complete resolution of the effects of the massive retrosternal goiter.

## Discussion

Retrosternal goiters are enlargements of the thyroid gland that extend into the mediastinum and account for approximately 5-10% of all goiters. They are clinically significant due to the potential for compressive symptoms, including tracheal deviation, dysphagia, or vascular compromise [3]. Giant recurrent retrosternal goiters are rare, and their management is particularly challenging because of distorted anatomy, scarring from previous surgery, and increased risk of injury to recurrent laryngeal nerves or parathyroid glands [4].

In this case, the patient presented with a massive recurrent retrosternal goiter causing tracheal deviation but without airway compression. This is in contrast to several reported cases where patients exhibited dyspnea, orthopnea, or dysphagia, highlighting the variability in clinical presentation [3]. The absence of compressive symptoms allowed for a cervical-only surgical approach, which has been demonstrated to be safe and effective when imaging confirms that the goiter does not extend below the thoracic inlet [5]. CT was crucial in preoperative planning, providing detailed information on the retrosternal extent, tracheal displacement, and relationship to mediastinal vessels, facilitating precise surgical dissection [3].

Recurrent retrosternal goiters are uncommon, especially after previous thyroid surgery, and increase the complexity of reoperative intervention. Scar tissue and altered anatomy elevate the risk of complications, including recurrent laryngeal nerve injury, hypocalcemia due to parathyroid damage, and bleeding [4]. Careful intraoperative identification and preservation of vital structures were paramount in this patient, resulting in an uneventful postoperative course with preserved voice function and stable calcium levels.

Histopathology confirmed a benign multinodular goiter with degenerative changes such as cystic areas, hemorrhage, and focal calcifications, consistent with long-standing nodular hyperplasia. Malignancy in retrosternal goiters is uncommon but has been reported in 10-15% of cases, reinforcing the role of surgical excision even in asymptomatic patients [5]. Surgical intervention not only alleviates potential compressive complications but also allows definitive histological diagnosis.

This case highlights that with meticulous preoperative imaging, careful surgical planning, and appropriate patient selection, even giant recurrent retrosternal goiters can be safely excised via a cervical approach with minimal morbidity. Importantly, this outcome was achieved in a low-volume surgical center, emphasizing that adherence to sound surgical principles, including detailed imaging assessment, cautious dissection in scarred planes, and preservation of the recurrent laryngeal nerves and parathyroid glands, can result in excellent outcomes beyond high-volume tertiary referral institutions. Regular follow-up with thyroid function testing and imaging is recommended to monitor for recurrence and ensure long-term outcomes [3-5].​​​​​​​

Key learning points include the rarity of giant recurrent retrosternal goiters, the utility of detailed imaging for surgical planning, the safety of cervical-only thyroidectomy in appropriately selected patients, and the importance of preserving recurrent laryngeal nerves and parathyroid glands to minimize postoperative complications.

## Conclusions

The present case demonstrates the successful management of a giant recurrent retrosternal goiter in a low-volume facility through a cervical approach with complete preservation of the recurrent laryngeal nerves and parathyroid glands. The patient recovered without complications and experienced full resolution of compressive symptoms. Careful preoperative assessment, detailed imaging, and coordinated multidisciplinary planning were central to achieving a safe and effective surgical result. This case reinforces several important lessons for clinical practice. Thorough evaluation and early recognition of progressive compressive symptoms are essential to prevent airway compromise and to guide timely referral for definitive surgical intervention. Even in reoperative settings, where fibrosis, adhesions, and distorted anatomy increase technical difficulty, meticulous dissection and close attention to critical structures can permit successful excision through a cervical route without resorting to more invasive procedures such as sternotomy.

The rarity of recurrent retrosternal goiters of this size, particularly in low-volume centers, underscores the value of documenting such cases to enhance understanding of optimal surgical strategies. Our findings support the growing evidence that a carefully planned cervical approach is adequate for many massive or recurrent retrosternal goiters when guided by comprehensive preoperative imaging and precise intraoperative technique. Continued reporting of similar cases will help refine management pathways and improve long-term outcomes for patients with complex thyroid disease.

## References

[1] Sridar K, Mohiyuddin SA, A S, Deo R, Mohammadi K, Raju K, Munireddy Papireddy S (2024). Outcomes of total thyroidectomy in large goiters with retrosternal extension and tracheal compression: a multivariate analysis. Cureus.

[2] Rui Sheng Y, Chong Xi R (2016). Surgical approach and technique in retrosternal goiter: case report and review of the literature. Ann Med Surg (Lond).

[3] Gupta H, Bhardwaj A, Bhardwaj V, Aggarwal V (2025). Retrosternal goiter: a mammoth hiding in plain sight — a case report. J Endocr Surg.

[4] Sorouri S, Akbarianrad S, Naseri M (2024). A case report of massive retrosternal goiter in a 54-year-old woman with symptoms of head and neck swelling and dyspnea. Clin Case Rep.

[5] Mohammad BH, Pusuluri M, Pandey S, Garg A, Saini R (2025). Huge multinodular goiter with retrosternal extension, a challenge to both surgeon and anesthetist: a case report. Int J Res Med Sci.

